# Protein Keeps Adjacent Tissues Growing in Synchrony

**DOI:** 10.1371/journal.pbio.1001006

**Published:** 2010-12-14

**Authors:** Richard Robinson

**Affiliations:** Freelance Science Writer, Sherborn, Massachusetts, United States of America

**Figure:**
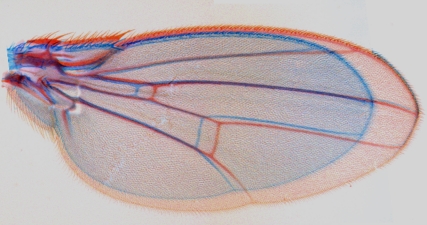
Recent work in the fruit-fly underscores a molecular mechanism by which adjacent tissues respond in a coordinated manner to local variations in growth and generate well-proportioned organs.

Why are hearts heart-shaped, and kidneys shaped like kidneys? More generally, what keeps multicellular creatures, and their individual parts, correctly proportioned even as they increase a thousand-fold or more in size? Despite the ubiquity of the phenomenon and the intriguing nature of the question, relatively little is known about the molecular mechanisms that regulate proportional growth. In this issue of *PLoS Biology*, Duarte Mesquita, Andres Dekanty, and Marco Milán offer one clue—identifying a protein that slows growth in one part of a fly wing when growth in another part is reduced.

Within the fly larva, cell clusters called imaginal discs develop as the larva grows. These clusters play no role in the larval stage of life; instead, they are the primordia of adult structures, including the wings. During metamorphosis, they reorganize, fuse, and grow to create the adult fly.

To examine the effect of growth retardation in one group of cells on the growth rate in another, the authors added the gene for several protein synthesis inhibitors, including the poison ricin, to one section (anterior or posterior) of the wing imaginal disc, leaving the other section untouched. The ricin gene’s expression could be turned off with cold temperatures and on with warm ones, and the larvae were initially grown in a cool room to allow normal wing disc development, then switched to a warm one, stunting growth.

They found that reducing growth in one section caused a similar reduction in the other section. When growth in the treated section was reduced more, by exposing the larvae to warm temperatures for longer, growth in the untreated section fell in lockstep. The result in all cases was a smaller, but still well-proportioned wing. There was no impact on other organs, or on the overall size of the fly.

The reduction in growth in the treated region (a so-called autonomous effect) was not surprising, since reducing protein synthesis reduces cell growth and proliferation. To explain the reduction in the neighboring region, a so-called non-autonomous effect, the authors first showed it was due mainly to a reduced number of cells, rather than a reduction in cell size, indicating an effect on mitosis or cell death.

They next examined the role of the protein dp53, a transcription factor and tumor suppressor known to regulate the cell cycle and apoptosis in times of stress. Reducing dp53 in the treated region had little effect on the final size of that section of the wing, consistent with the more profound negative effect of protein depletion on growth. But reducing dp53 in the treated region reversed the normally inhibited growth in the untreated region—when the activity or the quantity of the protein was reduced, the untreated wing region developed normally, despite the smaller size of the adjacent, treated, region, indicating a central role for dp53 in communicating between them. dp53 acted both directly on the adjacent tissue to reduce cell growth, and indirectly, through proteins called caspases, to reduce cell proliferation.

The results shed light on one mechanism for insuring development of a well-proportioned organ in a multicellular creature, and it is likely that p53, the mammalian form of dp53, plays a similar role in humans in the regulation of growth in closely adjacent tissues. Many mysteries remain, however. Coordinated growth over longer distances, such as between two limbs, will almost certainly involve multiple other mechanisms, and it will be interesting to see if p53 serves as a local enforcer of longer-range signals exchanged between more distant paired organs.

**Mesquita D, Dekanty A, Milán M (2010) A dp53-Dependent Mechanism Involved in Coordinating Tissue Growth in *Drosophila*. doi: 10.1371/journal.pbio.1000566**

